# Status of the Yellow Fever Inoculation

**Published:** 1887-10

**Authors:** J. McF. Gaston

**Affiliations:** Atlanta, Ga.


### STATUS OF THE YELLOW FEVER INOCULATION.

Dr. J. McF. Gaston, of Atlanta, Georgia, being called upon by the President of the Section on Hygiene of the Medical Congress to lead in the discussion of the paper on "Vaccination Against Yellow Fever," by Dr. Domingos Freire, of Rio De Janeiro, made the following remarks:

He feared that anything he might be able to say should lessen the favorable impression made upon the members of the section by the statistical data of Dr. Freire, indicating that out of 6,524 persons inoculated with the cultured virus of yellow fever during the years 1885 and 1886, only eight had died, while there had been about two thousand deaths in the same localities of those who had not been inoculated against the yellow fever.

He had been cognizant of the labors of Dr. Freire before leaving Brazil, and had kept up with his efforts since returning to this country. But the publications, made under the authority of the Brazilian Government, of all the details of the process and the results of the inoculation afford sufficient evidence of the trustworthiness of Dr. Freire's experiments to satisfy any unbiased mind. It must be understood that the names and residences of those inoculated being given, any one may verify the correctness of the records, and the opposition of a portion of the medical men and others in Rio to the work of Freire constitutes a severe test of his results. Like Jenner, who encountered the severest criticism of vaccinating against small-pox, Freire has had to overcome prejudice amongst his colleagues and the incredulous population of Rio De Janeiro. This affords a hope that something may be effected amongst us for the promotion of the protection against the greatest scourge of our Southern seaports.

When he became convinced, by the logic of facts, that the process of Freire afforded an immunity from attacks of yellow fever, it occurred to him that a statement of the results obtained by Freire would be likely to secure the prompt attention of the authorities of the United States Government. But on bringing this matter first to the notice of the Secretary of State and then to the attention of the President, and urging their early action in sending commissioners to investigate its claims, he was met with the reply that there were no funds set apart for such an object. He then enlisted the energetic co-operation of Dr. Joseph Holt, President of the Board of Health of New Orleans, who was further stimulated to urge this investigation by receiving about the same time a communication from Panama endorsing the efficacy of such inoculation as a protection against yellow fever.

His zealous advocacy of it before the American Association of Public Health at Washington two years ago led to the framing and presentation of a bill before Congress, authorizing the President to appoint three commissioners and providing for an appropriation of $30,000 to investigate yellow fever inoculation in Mexico and Brazil.

In the meantime a memorial was forwarded to both houses of Congress, in support of the bill then pending, by the American Medical Association, which met in St. Louis. But opposition was found from a quarter where it should have been least expected, and members of the profession connected with the New Orleans *Medical Journal* industriously circulated copies of it among the members of Congress, with articles intended to prejudice their minds against the passage of the bill. It is not desired to deprive them of any credit which may attach to their strenuous efforts to defeat the project of their colleague, Dr. Holt, but it is difficult to conceive that they were very anxious to promote the best interests of the population of that great city with which their lot is cast.

As a consequence of this and other opposition to the measure, a meagre appropriation of ten thousand dollars was made by Congress for the investigation of yellow fever inoculation in Mexico and Brazil.

Subsequently Dr. George M. Sternberg was appointed by the President as the sole commissioner to proceed with this work. Unfortunately, instead of being sent first to Mexico, having a summer corresponding with that of the United States, where he might have encountered cases of yellow fever in these months, he was ordered at once to Brazil, which, being in the Southern Hemisphere, has its winter at this time.

It was a further drawback to the objects in view that Dr. Domingos Freire, whose process was under consideration, should have been absent at that time from Rio. All who are conversant with French medical literature should have known, through the publications which appeared in regard to the reception extended to Dr. Freire in Paris, that he was occupied then and there with the demonstration of the claims of inoculation as a protection against yellow fever.

In consequence of this mistake, a month was spent in Rio by Dr. Sternberg without the assistance of Dr. Freire, and though he afterwards availed himself of his presence to go over with him the various processes employed in the attenuated cultures, yet there was no opportunity for a practical testing of yellow fever inoculation as a preventive measure.

No fault can perhaps be alleged against Dr. Sternberg, as he acted under the order of the United States Government.

The high claims of Dr. Sternberg as a bacteriologist did not afford a guarantee against miscarriage under such disadvantageous conditions for the performance of his work, and in anticipation of an untoward issue, he felt called upon at the Chicago meeting of the American Medical Association to ascertain the sense of the medical profession in regard to the appointment of two others on the commission, who should be competent to assist in the business details by an acquaintance with the climate and customs as well as the language of the respective countries. It was expected that Drs. John Guiteiras and H. M. Lane, who were named in the proposed memorial to the President, would fulfill all the requirements satisfactorily, and it is not supposed that with these two efficient assistants such mishaps could occur in future as in the past.

The resolutions recommending this addition to the commission were sustained by a full meeting of the Association, notwithstanding a motion by Dr. Rohe to lie on the table, and a motion subsequent to their passage for reconsideration. But on the following morning an unparliamentary proceeding was resorted to for rescinding the action, and the matter now stands in abeyance until there may be an occasion to secure again the free and independent expression of the American medical profession.

Realizing that the attitude of this section towards the further investigation of the prophylactic virtues of yellow fever inoculations must exert an important influence upon the medical world, whenever the proceedings of this Congress may be read, he had the honor to propose for consideration the following preamble and resolutions, which were unanimously adopted:

WHEREAS, Inoculation against yellow fever, if it proves successful after further examination, is calculated to benefit the human race throughout the world; and

WHEREAS, The facts presented by the experiments of Dr. Domingos Freire afford a reasonable assurance of its protective influence,

*Resolved*, That this section recommends the co-operative investigation of the results obtained by yellow fever inoculation as a protection against that disease, and that adequate appropriations by the governments represented in this Congress be made for that purpose.

*Resolved*, That this action be communicated forthwith for consideration in the general session of the Congress.

This action was subsequently endorsed by a vote of the Congress, and is therefore the authoritative voice of the medical profession of the world.

